# Practices related to sharps disposal among diabetic patients in Sri Lanka

**DOI:** 10.1186/s12930-018-0049-7

**Published:** 2018-12-07

**Authors:** K. R. Atukorala, S. I. Wickramasinghe, R. D. N. Sumanasekera, K. H. Wickramasinghe

**Affiliations:** 1 Department of Physiology, Faculty of Medical Sciences, University of Sri Jayewardenapura, Nugegoda, Sri Lanka; 2 Centre for Online Health, School of Medicine, University of Queensland, Brisbane, Australia; 3 General Practitioner, Colombo, Sri Lanka; 4 Senior Registrar in Ophthalmology, National Eye Hospital, Colombo, Sri Lanka

**Keywords:** Diabetes mellitus, Insulin, Sharps disposal

## Abstract

**Background:**

Patients with diabetes on insulin therapy use sharps (e.g., needles) on a regular basis and a considerable proportion of them, within their home environments. These sharps and other bloodstained materials, if not disposed of appropriately has the potential to be a public health hazard.

**Objective:**

Our objective was to explore the practices related to sharps disposal among patients with diabetes from North Colombo Teaching Hospital (CNTH), Ragama, Sri Lanka.

**Methods:**

We conducted a cross-sectional study on 158 patients with diabetes from the CNTH. Patients had to use sharps for the daily management of their disease for inclusion into the study group. Data were collected on sharps disposal practices using an interviewer-administered questionnaire. Clinic records were also used as a secondary data source.

**Results:**

Most patients, 153/158 (96.8%) used syringes to inject insulin. Forty-three patients (27%) involved others (e.g., family) when disposing of sharps. Used sharps were commonly disposed to the household garbage bin by 66 participants (41.7%). Other methods used for sharps disposal were: sharps container, toilet pit, household garbage dump and indiscriminate measures. Importantly most patients, 147 (93%) had received no information on how to dispose of sharps after usage.

**Conclusion:**

Patients commonly used unsafe practices in home-based sharps disposal. These included disposing of in the household garbage bin, burning sharps in the household garbage dump and disposing of into the common garbage dump of the community. Being male and being > 60 years of age was associated with a higher dependence on family members for sharps disposal. Patient education and public resources for sharps handling can help improve this situation.

## Introduction

Diabetes mellitus has grown into epidemic proportions across the world. Sri Lanka too is no exception to this epidemic and has witnessed significant increases in patient numbers throughout the country. At present, the total number of patients living with Diabetes in Sri Lanka is estimated to be around 2.8 million—approximately 13% of the population [1, 2]. Though the exact number of patients using Insulin in Sri Lanka is not known, the rapid rise in the number of patients living with diabetes in the country is most likely to have increased this number exponentially.

For many patients living with Diabetes, day to day control and management requires daily blood sugar measurements and insulin injections. These procedures invariably generate sharps within the household as most patients inject insulin at home. Improper disposal of these sharps has the potential to cause many public health problems [3–6]. These could include personal injury, blood-borne infections via needle stick injuries to others such as family members, neighbors and sometimes even the public.

Although the medical waste management and sharps disposal systems are well managed within the hospital settings in Sri Lanka, public health guidelines and public health services for home-based sharp disposal are currently unavailable. During preliminary scoping of public health literature and review of published peer-reviewed journal articles, we were unsuccessful in identifying published documents guiding proper sharps handling practices for patients in Sri Lanka.

## Objective

Our objective was to explore the practices related to sharps disposal among diabetic patients from North Colombo Teaching Hospital (CNTH), Sri Lanka.

## Methods

We conducted a cross-sectional study at the diabetes clinic of the CNTH from 15th May 2015 to 15th June 2015. The CNTH functions routine diabetes clinics and serves many patients daily. We were sampling from the main diabetic clinic. All diabetic patients on monthly routine review from the hospital were represented at this clinic.

We visited the clinic center at CNTH every working day of the week for one month to interview patients. The study population only included patients who were on routine review at the clinic. They were diagnosed of diabetes and were on routine clinic follow up. These patients were not acutely ill and were on monthly review to their diabetic clinic. The patients who were on insulin would come routinely every month to collect the free insulin.

We only included patients with Type 1 and Type 2 diabetes mellitus. Patients with gestational diabetes were excluded. All the patients had to be users of injectable insulin for at least a month’s duration. Both females and male adult patients of any age were included.

Each daily-clinic we visited served more than 50 patients each day. We selected every 3rd patient starting from number 1, from the clinic roster, using their ‘on the day’ clinic number.

In the event, a patient corresponding to the clinic number was not recruitable (not matching inclusion and exclusion criteria, patient in consultation with the medical officer, the patient with a member of the nursing team or the patient refused participation) we moved on to the next 3rd number. Some of the patients (every 3rd selected) were on oral hypoglycemic medication only and were excluded from selection.

We recruited, on average 6 to 8 patients daily. At the end of 1 month period, we had collected data from 158 patients. The number of patients seen daily at the clinic was not a constant. In the Sri Lankan context, some patients may decide to seek a review in the private sector occasionally and for the next visit may come to the government clinic for care. The clinic authorities only permitted us a month duration to enrol patients and we were time bound. Therefore we had to complete the data collection at the end of the month with the number we could collect only. Hence, we were only able to collect 158 patients during the collection time that was permitted. We did not keep exact records of the number of patients who refused participation, but the number of patients refusing to be interviewed was not many (as discussed at research group meetings with the data collectors). At the time the study was designed the primary research question was to identify how patients handled sharps and other Insulin-use related factors. Therefore, we have not delved into socio-economic considerations of patients. We assumed that patients might not be able to spend adequate time with data collectors due to the busy nature of the clinic and collection of detailed socio-economic and educational data would have necessitated us lengthening the questionnaire.

Information sheets and consent forms were provided for patients, and verbal consent was obtained before interviewing. Type of diabetes and duration of insulin treatment was confirmed by reviewing the clinic records with the patient’s permission. An interviewer-administered questionnaire was used to collect information. The structured questionnaire was developed using pre-identified questions from similar international journal articles [7, 8]. The issues explored insulin administration practices, equipment used, the frequency of needle use, disposal of insulin syringes and pens, lancet disposal, sharing of needles and knowledge about disease spread by sharing needles.

The questionnaire was pre-tested among a group of diabetic patients attending a family practice before the study. During pretesting we understood that patients have a “primary” method of disposal, i.e., how they ‘initially’ disposed of the sharps and a “secondary” mode of disposal—i.e., how they ‘ultimately’ got rid of it following the first disposal, e.g. the ones who disposed of sharps in the sharps bin (primary) later dumped the sharps bin to the garbage pits in their homes (secondary). The open-ended items in the questionnaire and direct interviews allowed the respondents to describe their actual needle disposal practices including ‘primary’ and ‘secondary’ disposal methods. The pre-trained research assistants (two pre-intern medical doctors) carried out the interviews. They were asked to carry out data collection at the pre-test of the questionnaire, and the data collection was found to be consistent and comparable among the two data collectors. Also, they were trained to follow the same sequence of questions in collecting data. Patient response was recorded in written without alteration by the interviewer to minimise interviewer bias. Daily patient records were collected and kept with a principal investigator (RDN) securely. Hard copy data were saved into an SPSS database. Descriptive statistics such as proportions, mean ± SD have been used to describe the data. Analysis of data was done using Chi squared test and Fisher’s Exact test at 95% confidence using Statistical Package for the Social Sciences-SPSS version 18. Ethical clearance was obtained from the Faculty of Medicine, University of Kelaniya (P/15/02/2014).

## Results

At the completion of data collection, 158 patients had been interviewed. Their ages ranged between 21 and 90 years of age with a mean age of 59.3 years (SD ± 10.23). Out of the 158 patients, 121 (76.6%) were female. Among the included patients, the mean duration of living with diabetes was 7.4 years (SD ± 5.09), and 155 (98.1%) of them were patients with type 2 diabetes. Insulin administration practices showed that the average duration of insulin use was 3.16 years (SD ± 3.88). Among these patients, 96 (60.7%) had used insulin for more than 1 year. Interestingly only very few patients; 5 (3.1%), used an Insulin pen as the majority were using syringes for daily injections. Most patients 132 (83.5%) had required more than two doses of Insulin per day. Out of the syringe users (153/158) many patients 150 (98%), had recapped and reused the same needle repeatedly. More than half of the syringe users 84/153 (54.9%) had used the same syringe 6 or more times, and 95/153 (62%) had used the same needle for 3 or more days (mean 6.29 times ± 4.8). Only 117/158 (74%) patients cleaned the injection site before the injection. Just 10 (6.3%) of the patients regularly checked blood sugar using needles. A large number; 73 (46.2%), involved others such as family members when injecting Insulin and 43 (27%) patients also included others in disposing of sharps.

Commonly generated sharps in the household were needles from syringes and needles from Insulin pens. These were disposed of into a typical household garbage bin by 66 (41.7%) patients. The other methods used to dispose of sharps were sharps container, toilet pit, common public garbage dump and indiscriminate methods (Table 1). Interestingly 15 (9.5%) of the patients had collected sharps since the beginning in plastic bags and has not got rid of them. Surprisingly when inquired it was found that they had not thought even about a plan of disposal.

**Table 1:** Primary and secondary sharps disposal methods within households

Primary disposal method	Frequency (n = 158)		Secondary disposal method	Frequency	
	Number	Percent %		Number	Percent %
Recap and dispose to the common household garbage bin	66	41.7	Home garbage pit/fire	23	14.6
			Disposed into the Garbage lorry of the local Municipal Council (MC)	43	27.2
Sharps container	9	5.7	Home garbage pit/fire	8	5.1
			Disposed in common garbage bin and then into garbage lorry of the MC	1	0.6
Toilet pit	8	5.1	N/A		
Paper bag	16	10.1	Common garbage dump of the area	14	8.9
			Common garbage bin and into MC garbage lorry	2	1.2
Burn (fire)	32	20.2	N/A		
Indiscriminate (loosely or no specific place)	11	6.7	N/A		
Collected since beginning in plastic bags without disposal	15	9.5	N/A		
Old well	1	0.6	N/A		

Eight patients (5.1%) disposed the sharps into a sharps container inside the home environment but later emptied it into the household garbage pit. One patient (0.6%) dumped the sharps container in the typical household garbage bin that was taken by the municipal workers. This must have been done without the knowledge of the municipal workers as they would not collect garbage with sharps if it were notified to them. In Sri Lanka, the municipality does not collect sharps. Importantly, none of the patients reported having used hospital facilities for sharps disposal. Thirty two (20%) patients burnt the sharps, while 8 (5.1%) dumped them in the latrine pits.

When age and gender were analyzed as essential variables in sharps disposal methods there was no statistical significance (Table 2). When comparing sharp disposal practices with the level of education, educational status was stratified as those who at least had ‘General certificate ordinary level qualification & above’ and those who were educated below the ordinary level. Qualifying this examination is a prerequisite for upper secondary education, and in Sri Lanka, this is considered the minimum requirement for entry into vocational training, or employment. Level of education higher than an ordinary level pass showed a unique use of safe primary disposal methods than the group who had less than an ordinary level pass {(Odds ratio) OR = 3.43 [95% (Confidence interval) CI 1.15–10.27], *p* = 0.04} Needles were shared by only one patient. It was when a male patient shared needles with his wife who herself was a diabetic—when checking blood sugar. Importantly, two patients reported that other family members had experienced accidental needle-stick injuries.

**Table 2:** Sharp disposal practices according to different characteristics of patients (*n* = 158)

(N = 158)	Practice of disposal of sharps			Seeking assistance with injections			Seeking help in disposal of sharps		
	Safe disposal value (%)	Unsafe disposal value (%)	*p* value	No value (%)	Yes value (%)	*p* value	No value (%)	Yes value (%)	*p* value
*Age*									
< 60	5 (3.2)	73 (46.2)	0.08	54 (34.2)	24 (15.2)	0.000*	64 (40.5)	14 (8.9)	0.01*
60 ≥	12 (7.5)	68 (43.0)		31 (19.6)	49 (31.0)		51 (32.2)	29 (18.3)	
*Gender*									
Male	6 (3.8)	31 (19.6)	0.22^a^	18 (11.4)	19 (12.0)	0.473	21 (13.3)	16 (10.1)	0.012*
Female	11 (6.9)	110 (69.6)		67 (42.4)	54 (34.2)		94 (59.4)	27 (17.0)	
*Level of education*									
Primary and lower secondary (< GCE O/L)	5 (3.2)	83 (52.5)	*0.0403*^a^*	38 (24.0)	50 (31.6)	0.004*	60 (37.9)	28 (17.7)	0.20
Upper secondary and tertiary (≥ GCE O/L)	12 (7.6)	58 (36.7)		47 (29.7)	23 (14.5)		55 (34.8)	15 (9.5)	
*Duration of use*, (years)									
< 1	8 (5.1)	19 (12.0)	*0.004*^a^*	12 (7.5)	15 (9.5)	0.517	18 (11.4)	9 (5.6)	*0.014* ^a^
1–5	7 (4.4)	99 (62.6)		60 (37.9)	46 (29.1)		84 (53.2)	22 (13.9)	
> 5	2 (1.3)	23 (14.6)		13 (8.2)	12 (7.5)		13 (8.2)	12 (7.5)	
*Prior education on safe disposal*									
Yes	6 (3.8)	5 (3.2)	*0.0002*^a^*	9 (5.6)	2 (1.2)	0.064^a^	11 (6.9)	0	*0.036*^a^*
No	11 (6.9)	136 (86.1)		76 (48.1)	71 (44.9)		104 (65.8)	43 (27.2)	

*GCE O/L* general certificate examination ordinary level

* Statistically significant

^a^Fishers exact test

Many patients 147 (93%) had never received any education regarding sharps disposal methods or the possibility of a blood-borne infection upon a needle stick injury. Patients who were educated about sharp disposal were likely to dispose of them “safely” (*p* < 0.001, OR 14.83, 95% CI 3.89–56.45).

Older patients (> 60 years of age) had a higher dependency on family members for injecting insulin (*p* < 0.001 OR 3.55; 95% CI 1.84–6.87) and in disposal of sharps (OR = 2.59 [95% CI 1.24–5.42], *p* = 0.04) (Table 2). Males involved family members more in disposing of sharps (OR = 2.65 [95% CI 1.21–5.78], *p* = 0.012). Patients with a lower level of education were seen to involve family members more to inject insulin (OR = 2.68 [95% CI 1.39–5.16], *p* = 0.004).

## Discussion

This is the first study done on this topic in Sri Lanka. We found that many of the interviewed patients handled household sharps poorly and disposed sharps either loosely or into the household common garbage bin, household garbage pit or a common garbage dump in the area. The findings are similar to other studies done in this regard [9–15]. Such unsafe practices pose a major threat to others through the possibility of needle stick injuries [12, 14]. Similar to other studies, the results show that most of the patients; 98% re-capped and re-used the same needle repeatedly [3, 15]. Furthermore > 50% of patients reported that they used the same needle over and over. This Re-capping and re-use of needles increase the risk of infection to the patient if proper procedures are not adhered to. The reuse of syringes has been consistently identified as a major route of hepatitis B and C transmission in other countries [4–6, 10]. Patients who were > 60 years of age were involving family members with injecting insulin and disposal of sharps. Male patients tended to involve family members in the disposal of sharps increasingly. This may be due to the traditional family structure in Sri Lanka, where the female tends to be responsible for activities in the kitchen which includes garbage disposal. It would be usual to expect patients with a higher level of education to be better able to cope with injecting insulin and handling sharps. As expected, lower level of education was associated with involving family members to inject insulin more.

In the event injections are administered by family members, re-capping and re-use create potential for needlestick injuries and the likelihood of spread of infections. Though only a few family members reported needlestick injuries, the actual number of injuries involving members of the immediate family may be higher.

When disposing of sharps, some patients tried to burn sharps, but needles and lancets cannot be incinerated by domestic fires; toxic fumes may be released in burning plastic syringes. The primary methods of disposing of sharps by the study group consisted of the sharps container and latrine pit. Although latter cannot be considered an ideal safe option, use of these methods showed that patients had some idea about keeping sharps away from others and could be described as generally ‘cautious or safe’ disposal methods. None of the secondary sharps disposal methods used by the study group could be described as safe. Patients who were educated about sharp disposal were likely to dispose of them “safely”. This finding is also supported by several other studies [3, 8, 9, 16, 17]. When all safe methods of sharps disposal are reviewed, there were no relationships between age, gender or type of diabetes. Those who injected insulin for less than 1 year had better sharp disposal practice than those who injected insulin for more than 1 year. This finding was in agreement with another study done in Philippines where longer duration of diabetes mellitus and insulin use negatively influenced disposal practices [18]. It may be because when one initially handles sharps they tend to be more cautious about it, but as they get used to it, it may be perceived as less threatening. However, in another study, insulin use more than 5 years, being type 1 had better practice [6]. These contradictory findings in different settings suggest that sharps disposal practises maybe more intrinsically related to motivation and encouragement of patients irrespective of the duration of insulin use. Being male gender, being > 60 years of age were associated with including a family member in injecting insulin and later in the disposal of sharps. Most adult patients tend to attend clinic appointments alone. Similar behavior is observed more in male patients. Even if advice on proper disposal of sharps had been provided during clinic time, the knowledge might not be transferred to family members at home.

As expected, patients with a higher level of education than ordinary level, successfully used several “safe” methods of primary disposal. This finding was in agreement with some studies [6, 8, 14, 18] while others did not support this [11]. However the higher educated are expected to have better ability to acquire information, so it is possible that they may have been better educated about their disease and to recognize the hazardous nature and need for safe disposal of their sharps. Even then, the sharps retainer that was used to collect sharps was dumped in garbage pits or disposed to the collection by the municipality, due to the lack of better options provided by the authorities. If the municipality workers were to know whether sharps were in the garbage, they collected they would refuse to collect it. This could be another reason why there were patients who had been using insulin for years but still had not disposed of their used sharps. These patients may have thought that it was not correct to put out sharps as it may harm others because in reality there were no safe methods available to them. Although a majority of patients 127 (80.4%) were aware of the spread of blood-borne infections through needlestick injuries, it had no bearing on safe, sharp disposal practices.

The study identifies two core issues that need urgent attention. Firstly, those patients who are conscious of the need for safe disposal of sharps were compelled to throw them into the general garbage collection eventually. This may be due to the unavailability of secure final disposal options like incinerators or government sharps collection schemes. Secondly, the possible lack of knowledge of available options for safe disposal of sharps among patients.

Designated sharps collection centers or coordinated community-level government mechanisms are currently unavailable in Sri Lanka. Disconcertingly, this deficiency may be a proxy of the lack of importance placed on this need by health authorities. Some countries practice distribution of hospital grade sharps collection bins to patients to use at home and once filled are collected at the hospital [12, 19]. Similar methods may be useful in the Sri Lankan context, and some patients may even wish to purchase these if made available commercially.

At present government hospitals in Sri Lanka provide insulin products free of charge for patients. Sharps collection boxes, similarly, could also be provided to the patients with their medications. Though, disposal of sharps in health care settings in Sri Lanka is well regulated no such regulation is available for residential settings. In comparison, in some developed countries, strict guidelines on domestic sharps disposal practices are in a place where patient support schemes mandatorily provide domestic sharps boxes and later collect them at designated points. Severe penalty/fines are imposed on patients who do not corporate with the guidelines. Options such as community drop off programs, syringe exchange programmes, sharps mail-back programs, residential waste special pick-up programs for sharps or at home needle destruction devices (needles are burnt or melt rendering it safe for disposal) are also reported from some developed countries [19–21]. In some countries, safe sharps website, smartphone-based applications have been created for the guidance of patients in finding the location of secure disposal services [22]. Though Sri Lanka may lack the financial resources for such enhanced systems, simple solutions such as using a “hard plastic” bottle to collect sharps at home could be implementable widely [23]. These could be later collected at hospital clinics or designated counters. Commercial providers could also be encouraged to provide safe sharps disposal options in public places (e.g., bus stops) as a longer-term solution.

The study shows that only a few of the recruited patients had been educated regarding methods of correct disposal of sharps. This can be considered an opportunity lost as the majority who received information on disposal found to have disposed of sharps cautiously. Education of patients has the potential to minimise hazards to patients themselves and also to family members. Methods to educate Sri Lankan patients can include the use of informational videos, supplemental reading material and teaching sessions during clinic time. The clinicians need to encourage their patients to use and dispose of sharps safely, and these clinical interactions can be a good point of contact for education as it can encompass full coverage of all types of patients with Diabetes. Patients, their family members, and local healthcare workers need to be educated on the importance of proper disposal of sharps. Local healthcare workers, especially of the public health service can be requested to propagate the message to families. Locally available resources such as a local hospital can be asked to open-up the sharps handling program to the general public of the area. Identifying cost-effective methods in disposing of sharps is also needed for the longer term.

The limitations of this study include having only a limited number of patients, poor collection of data on ethnicity, religion and socio-demographic data and being a single center study. Furthermore, very few patients had used insulin pens. In the private health sector in Sri Lanka, most patients use insulin pens as affordability may not be an issue as with government sector patients. Therefore future research should be multi-centered to include all strata of the insulin-using patient population. The questionnaire was not validated and is identified as a limitation.

Interviews were conducted by two interviewers so ‘Observer bias’ may have played a part in data diversity.

Some respondents may have had difficulty in reporting events that happened in the past (e.g., duration of insulin therapy, diagnosis of diabetes, etc.), so the potential for recall bias must also be considered as a limitation. Also, the Study could have been improved if answers to following questions were also sought. Whether they inject insulin only at home or even at work? How they dispose of when they are away from home? Among the patients who were said to be educated about sharp disposal—who provided education? Also, the reasons for the current practice could have been asked. Still, the results available will be useful in providing information for further study.

## Conclusion

The study shows that a majority of the patients interviewed, disposed of sharps using indiscriminate and potentially hazardous means. A feasible method that could be proposed for immediate implementation in Sri Lanka is to encourage the patients to handover their sharps retainer to the hospital during their monthly clinic visits, just before obtaining the free consignment of insulin. Public willingness to pay for sharps disposal services or desire to pay extra for insulin products where the pharmaceutical provider will bear the responsibility of collecting and properly disposing of sharps can also be explored. Education of patients living with diabetes regarding acceptable methods of disposal of sharps via patient education programs and even via development of national guidelines is hence, essential and urgent. Following implementation of national guidelines, a penalty system could also be introduced where inappropriate disposal will be fined or penalized. Developing publicly placed resources for the disposal of sharps too needs urgent consideration by authorities.
